# *Toxocara canis* mucins among other excretory-secretory antigens induce in vitro secretion of cytokines by mouse splenocytes

**DOI:** 10.1007/s00436-015-4561-5

**Published:** 2015-06-06

**Authors:** Ewa Długosz, Katarzyna Wasyl, Maciej Klockiewicz, Marcin Wiśniewski

**Affiliations:** Division of Parasitology, Department of Preclinical Sciences, Faculty of Veterinary Medicine, Warsaw University of Life Sciences, Ciszewskiego 8, 02-786 Warsaw, Poland; W. Stefański Institute of Parasitology, Twarda 51/55, 00-818 Warsaw, Poland

**Keywords:** *Toxocara*, Excretory-secretory antigens, Mucins, Cytokines

## Abstract

The effect of *Toxocara* larval antigens on cytokine secretion by mouse splenocytes was studied in vitro. Recombinant mucins were produced in *Pichia pastoris* yeast, and *Toxocara* excretory-secretory (TES) antigens were collected from in vitro culture of L2 larvae. *Tc*-MUC-2, *Tc*-MUC-3, *Tc*-MUC-4, and *Tc*-MUC-5 were expressed as glycoproteins and were specifically recognized by *Toxocara canis*-infected dog serum antibodies. Mouse splenocytes stimulated with recombinant mucins produced IL-5, IL-6, and TGF-β. Cell stimulation with whole TES products was more effective and resulted in secretion of IL-4, IL-5, IL-6, IL-10, and TGF-β and downregulation of TNF-α production. IFN-γ and IL-17 secretion was noted only after ConA treatment. Cells originating from infected animals produced significantly smaller amounts of these two cytokines compared to control cells, which suggests that Th1 and Th17 response in infected mice is strongly inhibited. However, splenocyte stimulation with both TES and ConA upregulated the production of IFN-γ and IL-17. This shows that TES antigens have strong immunomodulatory properties and are able to induce a broad range of effects on murine immune cells.

## Introduction

*Toxocara canis* is a common nematode parasite of dogs. Embryonated eggs are infective to a wide range of mammal hosts. Human toxocariasis is a zoonotic disease prevalent worldwide (Despommier 2003). Larvae migrate through tissues for months up to several years evading the immune attack of the host. They reach several organs such as the lung, liver, eye, and brain causing severe complications. *Toxocara* worms also contribute to the development of allergic diseases, including asthma (Pinelli et al. 2008).

Infective larvae release significant amounts of *Toxocara* excretory-secretory (TES) antigens at around 1 % of their body weight per day (Meghji and Maizels 1986). The major excretory-secretory macromolecules are all glycoproteins which differ in essential characteristics. TES-120 fraction contains *O*-glycosylated mucins (Gems and Maizels 1996; Loukas et al. 2000b), and TES-32 and TES-70 have been identified as C-type lectins *Tc*-CTL-1 (Loukas et al. 1999) and *Tc*-CTL-4 (Loukas et al. 2000a) and TES-26 as a phospohatidylethanoloamine-binding protein (Gems et al. 1995). The major role of TES-120 mucins is the formation of larval surface coat (Page et al. 1992). They protect the parasite from antibody and eosinophil attack, as the entire coat is shed upon binding of these molecules (Smith et al. 1981; Badley et al. 1987). *O*-methylated *Toxocara* glycans are specific targets for host antibodies which proves they are strongly recognized by the host immune system (Schabussova et al. 2007). However, the influence of secreted mucins on the cellular immune response has not been studied yet.

This work confirms previous observations that in the experimental model of murine toxocariasis infection induces a strong Th-2-like response with increased IL-10 and TGF-β production (Kuroda et al. 2001; Fan et al. 2004; Wu et al. 2008; Faz-López et al. 2013). We also report that despite the presence of IL-6 and TGF-β which are key cytokines in Th17 differentiation (Basso et al. 2009), this type of immune response is not induced during *T. canis* infection in mice. Production of cytokines by mouse splenocytes can be stimulated with recombinant *Toxocara* mucins, but whole TES products have bigger impact on cytokine secretion by these cells. Whether this effect is attributable to some other TES component or is a sum of effects of all particular TES proteins remains unclear.

## Materials and methods

### Preparation of *Toxocara* ES antigens

Adult *T. canis* worms were collected from feces of dewormed dogs treated in veterinary clinics in Warsaw. Eggs were obtained from dissected female worms and incubated in 0.1 N H_2_SO_4_. Fully embryonated and infective eggs were hatched as described by Oaks and Kayes (1979) and maintained in vitro in Minimal Essential Medium (Sigma-Aldrich) supplemented with penicillin (100 U/ml), streptomycin (100 μg/ml), and amphotericin B (2.5 μg/ml) at 37 °C, 5 % CO_2_. Culture medium was replaced every 3 days, and the spent medium was collected, concentrated, and dialysed against sterile phosphate-buffered saline (PBS) using Amicon Ultra Centrifugal Units (Millipore). TES solution was filtered through 0.22-μm filter, and antigen concentration was determined using BCA Protein Assay (Thermo Scientific).

### Production of recombinant mucins

Total RNA was isolated from *T. canis* larvae and reverse transcribed into cDNA which was used as a template for amplification of fragments encoding mucins (*Tc-muc*-2, *Tc-muc*-3, *Tc-muc*-4, and *Tc-muc*-5). Primers were designed basing on sequences cloned by Tetteh et al. (1999) and Doedens et al. (2001) and published in GenBank (accession numbers: AF167707, AF167708, AF167709, and AF167710). First, full coding sequences were cloned into pGEM-T Easy vectors (Promega), and then, fragments lacking signal peptide sequences were amplified and cloned in frame into pPICZαA vectors (Life Technologies) using EcoRI and XbaI restriction sites. Primer sequences were as follows: *Tc-muc-2*-F: 5′ CCG GAA TTC CAG CCT GGT GCC CAA 3′, *Tc-muc-2*-R: 5′ GCT CTA GAG CAC AGA AGC CGC ACG TCA GTG 3′, *Tc-muc-3*-F: 5′ CCG GAA TTC CAATCGATATTCGCA 3′, *Tc-muc-3*-R: 5′ GCT CTA GAG CCG AAC AAA AAC CGC ACG ACA 3′, *Tc-muc-4*-F: 5′ CCG GAA TTC GCTACAACAACAACT 3′, *Tc-muc-4*-R: 5′ GCT CTA GAG CAC AGA AGC CGC ACG TCA GTG 3′, *Tc-muc-5*-F: 5′ CCG GAA TTC AGCACTACAGCTGTA 3′, *Tc-muc-5*-R: 5′ GCT CTA GAG CAC ACA AAG CAC ACG TCT TCT 3′. Positive pPICZαA clones were verified by nucleotide sequencing. Recombinant plasmids were then linearized and transformed into *Pichia pastoris* X33 strain using Pichia Easy Comp Kit (Life Technologies).

Expression of recombinant mucins was performed in Buffered Minimal Methanol Medium at 28 °C for 96 h. Proteins were purified from culture media using HIS-Select HF Nickel Affinity Gel (Sigma-Aldrich). Eluted fractions were concentrated and dialysed against sterile PBS with Amicon Ultra Centrifugal Units (Millipore). The presence of purified recombinant mucins was confirmed by SDS-PAGE and Western blotting analysis. The presence of glycan moieties was confirmed by staining with Pierce Glycoprotein Staining Kit (Thermo Scientific).

### Western blotting

Recombinant mucins were separated by SDS-PAGE using 12.5 % polyacrylamide gel and transferred onto nitrocellulose membrane. The membrane was blocked in 2 % skimmed milk in PBS buffer, followed by incubation with horseradish peroxidase (HRP)-conjugated monoclonal anti-polyhistidine antibodies (Sigma-Aldrich) or *T. canis*-infected dog sera (1:1000) obtained from the Veterinary Medicine Faculty Clinic at Warsaw University of Life Sciences-SGGW. The reaction was detected with HRP-conjugated rabbit anti-dog IgG antibodies (Jackson ImmunoResearch). Membranes were developed with SuperSignal West Pico Chemiluminescent Substrate (Thermo Scientific) on Kodak films.

### Mice and experimental *T. canis* infection

Eight-week-old male BALB/c mice (*n* = 3) were orally infected with 500 *T. canis* infective eggs. Non-infected mice (*n* = 3) were used as controls. During the experiment, animals were maintained in standard conditions, exposed to 12-h light-dark cycles, and provided with water and food ad libitum. At day 21 p. i. animals were euthanized. Two independent experiments were performed.

### In vitro cell culture

Spleens from one experimental group were pooled and cell suspensions were prepared. Erythrocytes were lysed with Red Blood Cell Lysis Buffer (Sigma-Aldrich). The suspension was depleted of monocytes/macrophages with CD11b MicroBeads (Miltenyi Biotec) which were used in a different experiment. Remaining cells were suspended in RPMI 1640 supplemented with 10 % FBS, penicillin, and streptomycin (100 U/ml and 100 μg/ml) (all from Sigma-Aldrich) and cultured at 1.5 × 10^6^/ml in 24-well culture plate at 37 °C in 5 % CO_2_. Cells were stimulated for 24 h with 5 μg/ml of *Toxocara* ES antigens, 5 μg/ml of recombinant mucins (*Tc*-MUC-2, *Tc*-MUC-3, *Tc*-MUC-4, and *Tc*-MUC-5–1.25 μg/ml each), and 5 μg/ml of ConA. Culture supernatants were harvested and used for cytokine assays.

### Cytokine assay

IL-4, IL-5, IL-6, IL-10, IFN-γ, and TNF-α concentrations in culture supernatants were determined using BD Opt EIA ELISA Sets (BD Biosciences) and IL-17 and TGF-β using DuoSets (R&D Systems) according to manufacturer instructions.

### Statistical analysis

Statistical analysis was performed by Student’s *t* test. A value of *P* < 0.05 was considered to be significant. Analysis was done using Statgraphics Plus 4.1 software. Results are shown as mean ± SD of quadruplicate cultures in one experiment.

## Results

### Production of recombinant mucins

**Fig. 1:**
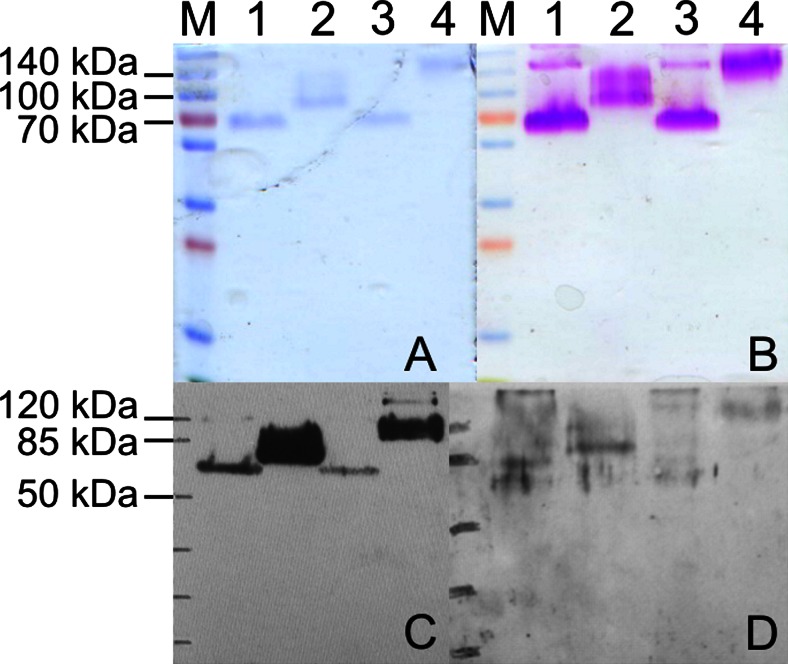
SDS-PAGE (**a**, **b**) and Western blotting (**c**, **d**) analysis of *Toxocara* recombinant mucins produced in *Pichia pastoris*. 5 μg of *Tc*-MUC-2 (*lane 1*), *Tc*-MUC-3 (*lane 2*), *Tc*-MUC-4 (*lane 3*), and *Tc*-MUC-5 (*lane 4*) were separated on 12.5 % polyacrylamide gels and stained with Coomassie Blue (**a**), Glycoprotein Staining Kit (**b**), transferred on nitrocellulose membranes, and detected with monoclonal anti-polyhistidine antibodies (**c**) or *T. canis*-infected dog serum IgG antibodies (**d**) (blotting analysis with one of three infected sera is shown)

SDS-PAGE analysis of purified recombinant mucins showed single bands of 70 kDa for *Tc*-MUC-2 and *Tc*-MUC-4, 100 kDa for *Tc*-MUC-3, and more than 120 kDa for *Tc*-MUC-5 (Fig. 1a). Glycoprotein staining showed intensive glycosylation of all expressed mucins (Fig. 1b). The presence of glycan moieties on recombinant mucins was also confirmed by lectin-binding assay which showed that concanavalin A (ConA) binds to all four glycoproteins (data not shown). Recombinant mucins were specifically recognized by anti-HIS-tag (Fig. 1c) and serum antibodies of *T. canis*-infected dogs (Fig. 1d). Control uninfected dog serum did not react with investigated antigens (data not shown). Additionally, different recombinant proteins produced in *P pastoris* X33 strain were also tested as controls. Two *Fasciola hepatica* and one *Hypoderma diana* recombinant antigens were used. Antibodies from *T. canis*-infected or control dogs did not recognize these proteins (data not shown).

### Cytokine production by mouse splenocytes

**Fig. 2:**
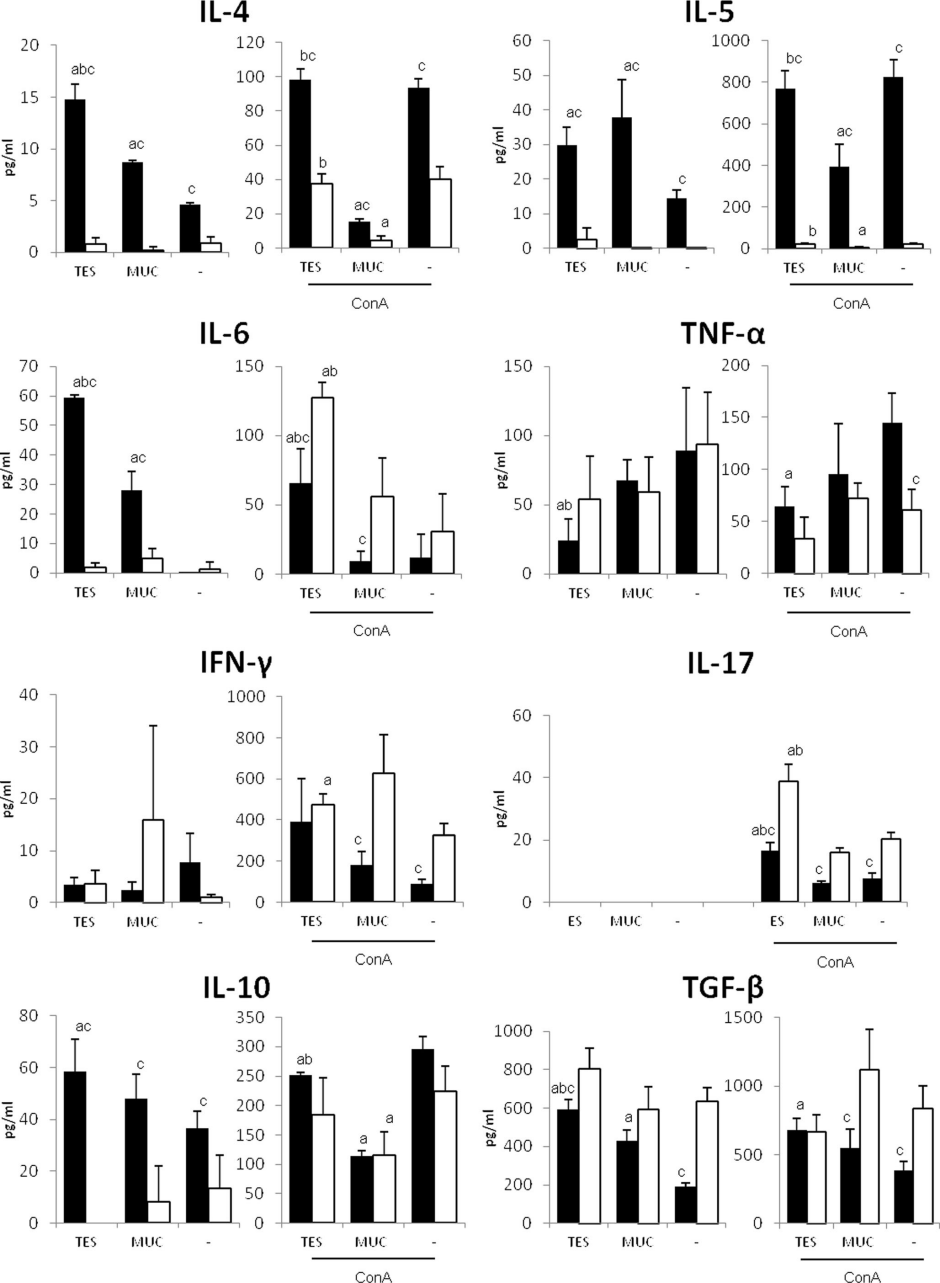
Cytokine production by splenocytes from *T. canis*-infected (*black bar*) and control (*white bar*) mice. Mice were infected orally with 500 embryonated eggs for 21 days. Spleen cells (1.5 × 10^6^/ml) depleted of monocytes/macrophages were stimulated in vitro for 24 h with TES (5 μg/ml), recombinant mucins (MUC: *Tc*-MUC-2, *Tc*-MUC-3, *Tc*-MUC-4, and *Tc*-MUC-5 1.25 μg/ml each) or unstimulated (*solid line*) alone or in combination with ConA (5 μg/ml). Each group consisted of three mice, and spleen cells from three mice were pooled and cultured in quadruplicates. Results are expressed as mean ± SD in one of two independent experiments. Statistical analysis was performed by Student’s *t* test. A value of *P* < 0.05 was considered to be significant. *a* significantly different from unstimulated cells; *b* significantly different from MUC-stimulated cells; *c* significantly different from the corresponding cells from uninfected mice

As shown in Fig. 2, splenocytes from *T. canis*-infected mice produced significant amounts of IL-4, IL-5, IL-6, and IL-10 after stimulation with TES or recombinant mucins. TGF-β secretion was also stimulated by larval antigens although its concentration was lower compared to control group. On the contrary, secretion of TNF-α was inhibited by TES antigens but not by mucins. Production of IFN-γ and IL-17 was noted only after simultaneous stimulation with antigens and ConA. Interestingly, splenocytes from infected mice produced lesser amounts of IL-6, IL-17, and IFN-γ compared to normal mice. Again, TES antigens potentiated the production of these proinflammatory cytokines and inhibited TNF-α release by ConA-treated cells from infected as well as control mice. IL-4, IL-5, and IL-10 secretion by ConA-stimulated cells was not influenced by larval antigens, but lower concentrations were noted after stimulation with mucins in combination with ConA.

## Discussion

*P. pastoris* has been successfully used for production of many parasitic antigens (Bąska et al. 2013a; Bąska et al. 2013b; Rogé et al. 2013; Zawistowska-Deniziak et al. 2013) including *T. canis* TES-120 (Fong and Lau 2004). This expression system is highly effective, secretion of recombinant proteins into the medium simplifies affinity purification and what is the most important *Pichia* yeast carry out both *O*- and *N*-glycosylation (Ahmad et al. 2014). It has been well documented that helminth glycans induce Th2 and regulatory type immune responses via C-type lectin receptors on antigen presenting cells (reviewed by Thomas and Harn 2004; Prasanphanich et al. 2013). Therefore, generation of antigens in their glycoprotein form is crucial for determination of their impact on the immune response of the host. That cannot be accomplished while expressing parasitic antigens in bacterial systems.

*Toxocara* mucins are members of TES-120 family of *O*-glycosylated proteins secreted by this parasite. They are major components of larval surface coat (Page et al. 1992). *Tc*-MUC-1, *Tc*-MUC-2, and *Tc*-MUC-3 are also secreted together with other ES antigens (Gems and Maizels 1996; Loukas et al. 2000b). *Tc*-MUC-5 is larger and more divergent than the other mucins suggesting that it is not part of the TES-120 family of surface coat proteins (Doedens et al. 2001). Although on SDS-PAGE mucins form a closely migrating set of 120 kDa, their actual molecular weight is smaller as determined by mass spectrometry. This difference is attributable to high carbohydrate content. The real mass of *Tc*-MUC-1 is 39.7 kDa, *Tc*-MUC-2 is 47.8 kDa, and *Tc*-MUC-3 is 45 kDa (Loukas et al. 2000b).

Molecular masses of recombinant mucins generated in *P. pastoris* were approximately 70 kDa for *Tc*-MUC-2 and *Tc*-MUC-4 and 100 kDa for *Tc*-MUC-3. This is in line with results obtained by Loukas et al. (2000b) who showed that antibodies raised to recombinant *Tc*-MUC-3 expressed in *Escherichia coli* specifically recognize a higher molecular weight band of the TES-120 family. As predicted, *Tc*-MUC-5 molecular mass was the highest, although difficult to estimate, as the band was located between 120 and 250 kDa marker bands. The presence of glycans on recombinant *Toxocara* mucins was confirmed by staining with glycoprotein staining reagents and with ConA binding assay. These antigens were recognized by antibodies from *Toxocara*-infected dog sera, which proves that their structure did not significantly differ from native glycoproteins.

*Toxocara* larvae release significant amounts of heavily glycosylated excretory-secretory antigens, among which mucins and C-type lectins are the most abundant (Tetteh et al. 1999). These products are likely to play an important role in immune evasion. TES-32 and TES-70 components of *Toxocara* excretions have been identified as C-type lectins *Tc*-CTL-1 (Loukas et al. 1999) and *Tc*-CTL-4 (Loukas et al. 2000a). It is postulated that C-type lectins from tissue-dwelling nematodes might interfere with infiltration of leukocytes by competitively inhibiting selectin-mediated inflammation (Loukas and Maizels 2000). The surface coat composed of mucins is used to physically escape from the immune attack of the host. *T. canis* larvae shed the entire glycoprotein layer in response to binding by antibodies (Smith et al. 1981) or eosinophils (Badley et al. 1987). Dent et al. (1999) also confirmed that *T. canis* larvae are not susceptible to the actions of eosinophils as intensive eosinophilia had little impact on larvae survival and migration in IL-5 transgenic mice.

The aim of this study was to evaluate the impact of *Toxocara* mucins on the cellular immune response of the host. There are many reports on TES influence on cytokine secretion during *Toxocara* infection, but the separate role of mucins or other TES components in this process has not been determined yet. Spleen cells stimulated with recombinant mucins secreted significant amounts of IL-5, IL-6, and TGF-β. Surprisingly, concentration of IL-4, IL-5, and IL-10 was the lowest when cells were treated simultaneously with mucins and ConA. This downregulation however cannot be attributed to the primary function of parasite mucins. We proved that ConA binds to recombinant glycoproteins. Therefore, the potent stimulatory effect of ConA on splenocytes is most probably inhibited by bound mucin glycans. Such effect was not observed when splenocytes were stimulated with TES in combination with ConA.

However, cell treatment with TES products which also contain secreted mucins was more effective as the production of cytokines was higher. In response to TES cells also secreted IL-10, and after additional treatment with ConA, they produced IL-17 and IFN-γ. These results suggest that besides mucins some other components of *Toxocara* ES products have a strong impact on cytokine production.

*T. canis* larvae induce strong Th2 response with IL-4 and IL-5 production (Inuo et al. 1995; Lin et al. 2008; Malheiro et al. 2008; Faz-López et al. 2013), but regulatory cytokines are also produced (Kuroda et al. 2001; Fan et al. 2004; Malheiro et al. 2008). Th1 response is strongly inhibited, as production of IFN-γ and IL-12 is reduced (Kuroda et al. 2001; Malheiro et al. 2008).

Our results confirm previous observations. Spleen cells from *T. canis*-infected mice secrete significant amounts of IL-4, IL-5, and IL-10, and their production is upregulated by restimulation with TES or recombinant mucins. We noted lower TGF-β levels in infected mouse splenocyte cultures compared to controls, but after treatment with parasite antigens, the production of this cytokine increased. Our experiment was conducted 3 weeks p. i. In infected organs such as the liver, TGF-β production by leukocytes infiltrating the inflammatory lesions is observed from 4 weeks p. i. and increases up to 16 weeks p. i. (Wu et al. 2008). At the same time, Foxp3-expressing cells can be detected in the liver of infected mice (Othman et al. 2011); it is therefore possible that they are the source of TGF-β. The same experiment also showed that Foxp3 mRNA expression in the spleen significantly increases since 5 weeks p. i.

Production of regulatory cytokines by splenocytes from *Toxocara*-infected mice is correlated with weak secretion of proinflammatory cytokines after ConA treatment. Concentration of IL-6, IFN-γ, and IL-17 in these cultures was significantly reduced compared to cell cultures from control mice. On the other hand, TES antigens upregulate ConA stimulated production of these cytokines even in cells from control mice. Another proinflammatory cytokine, TNF-α is downregulated by TES which was also observed by Kuroda et al. (2001). This proves that the immune response is strongly regulated in infected animals and that TES products perhaps play a key role in this process. Such state of immune incompetence is probably long lasting as reinfection with *Toxocara* larvae favors parasite migration and survival (Kolbeková et al. 2011).

Strong Th2 response and production of regulatory cytokines during *Toxocara* infection in paratenic hosts lead to suppression of Th1 response with IL-12 and IFN-γ downregulation. Our studies showed that IFN-γ is secreted by mouse spleen cells only after ConA stimulation and that the concentration of this cytokine is significantly lower in cultures of cells from infected animals. Decreased levels of INF-γ are likely to be responsible for larval transmission from pregnant bitches to their puppies (Torina et al. 2005). IFN-γ production increases in older puppies in which the infection is controlled. Similar correlations between IFN-γ levels and parasite burdens have been observed in case of other helminth parasites such as *Schistosoma mansoni* (Correa-Oliveira et al. 2000), *Ascaris lumbricoides*, and *Trichuris trichura* (Geiger et al. 2002) and hookworms (Quinnell et al. 2004; Długosz et al. 2010). Indeed, in *Toxocara* infections, a weak Th2 type response results in limitation of larvae counts, but on the other hand, it leads to exacerbation of the immunopathology caused by the parasite in the lungs (Faz-López et al. 2013).

IL-6 is a cytokine with a wide range of functions, one of which is the regulation of acute and chronic inflammation (Naka et al. 2002). Together with TGF-β, it also plays a pivotal role in favoring Th17 differentiation (Basso et al. 2009). We have noted significant increase in IL-6 secretion by spleen cells from *Toxocara*-infected mice after stimulation with TES or recombinant mucins. Upregulated production of IL-6 by peritoneal macrophages isolated from infected mice was also reported by Kuroda et al. (2001). Our results show that IL-6 produced in response to *Toxocara* antigens does not induce the differentiation of Th17 cells. IL-17 was only produced when spleen cells were stimulated with ConA; moreover, the amount of IL-17 produced by cells from infected mice was twofold lower compared to controls.

New insights into the role of IL-6 in helminth infections have recently been reported by Smith and Maizels (2014). IL-6-deficient mice infected with *Heligmosomoides polygyrus* mounted stronger adoptive Th2 response, and worm expulsion was potentiated in these animals. This experiment led to the conclusion that in vivo IL-6 limits the Th2 response by modification of Treg-cell phenotype and promotes host susceptibility following helminth infection. It seems unlikely that IL-6 plays the same role during *Toxocara* infection. In this case, limitation of the Th2 response is not favorable to the parasite as the infection is efficiently controlled; it is also not favorable to the host as the tissue pathology is more intense (Faz-López et al. 2013). Othman et al. (2011) showed that Treg cells take part in the immune response against *Toxocara* larvae. Likely, IL-6 controls their development, but still little is known about the role of these cells in toxocariasis. The production of the hallmark regulatory cytokines IL-10 and TGF-β after contact with TES or mucins again implies the role of Treg cells in *Toxocara* infection.

We have shown that recombinant *Toxocara* mucins stimulate IL-5, IL-6, and TGF-β production by mouse splenocytes, but in a smaller degree than whole TES antigens. TES induce secretion of Th2 and regulatory cytokines, and they upregulate proinflammatory IL-6, IFN-γ, and IL-17 production by ConA treated cells and downregulate TNF-α production. This proves that TES antigens have potent immunoregulatory properties.
